# A Distinct Urinary Biomarker Pattern Characteristic of Female Fabry Patients That Mirrors Response to Enzyme Replacement Therapy

**DOI:** 10.1371/journal.pone.0020534

**Published:** 2011-06-15

**Authors:** Andreas D. Kistler, Justyna Siwy, Frank Breunig, Praveen Jeevaratnam, Alexander Scherl, William Mullen, David G. Warnock, Christoph Wanner, Derralynn A. Hughes, Harald Mischak, Rudolf P. Wüthrich, Andreas L. Serra

**Affiliations:** 1 Division of Nephrology, University Hospital, Zürich, Switzerland; 2 Division of Nephrology and Hypertension, University of Miami Miller School of Medicine, Miami, Florida, United States of America; 3 Mosaiques Diagnostics and Therapeutics AG, Hannover, Germany; 4 Division of Nephrology, University Hospital, Würzburg, Germany; 5 Department of Haematology, Hampstead Campus, University College Medical School, London, United Kingdom; 6 Proteomics Core Facility, Faculty of Medicine, University of Geneva, Geneva, Switzerland; 7 School of Life Sciences, College of Medical, Veterinary and Life Sciences, University of Glasgow, Glasgow, United Kingdom; 8 Department of Medicine, University of Alabama at Birmingham, Birmingham, Alabama, United States of America; 9 BHF Glasgow Cardiovascular Research Centre, University of Glasgow, Glasgow, United Kingdom; Stanford University School of Medicine, United States of America

## Abstract

Female patients affected by Fabry disease, an X-linked lysosomal storage disorder, exhibit a wide spectrum of symptoms, which renders diagnosis, and treatment decisions challenging. No diagnostic test, other than sequencing of the alpha-galactosidase A gene, is available and no biomarker has been proven useful to screen for the disease, predict disease course and monitor response to enzyme replacement therapy. Here, we used urine proteomic analysis based on capillary electrophoresis coupled to mass spectrometry and identified a biomarker profile in adult female Fabry patients. Urine samples were taken from 35 treatment-naïve female Fabry patients and were compared to 89 age-matched healthy controls. We found a diagnostic biomarker pattern that exhibited 88.2% sensitivity and 97.8% specificity when tested in an independent validation cohort consisting of 17 treatment-naïve Fabry patients and 45 controls. The model remained highly specific when applied to additional control patients with a variety of other renal, metabolic and cardiovascular diseases. Several of the 64 identified diagnostic biomarkers showed correlations with measures of disease severity. Notably, most biomarkers responded to enzyme replacement therapy, and 8 of 11 treated patients scored negative for Fabry disease in the diagnostic model. In conclusion, we defined a urinary biomarker model that seems to be of diagnostic use for Fabry disease in female patients and may be used to monitor response to enzyme replacement therapy.

## Introduction

Fabry disease (OMIM 301500) is a rare X-linked inherited lysosomal storage disorder caused by deficient enzymatic activity of α-galactosidase A (GLA). The resulting defect in the catabolism of α-D-galactosyl-containing compounds leads to intracellular accumulation of glycosphingolipids, particularly globotriaosylceramide (Gb_3_), causing progressive cellular dysfunction. Main clinical manifestations include: renal disease characterized by proteinuria along with progressive decline of glomerular filtration rate (GFR), left ventricular hypertrophy, stroke, acroparesthesia, hypohidrosis, corneal opacities and angiokeratomas.

Women were previously considered to be mostly asymptomatic carriers of the disease with mild clinical features. However, it has recently become evident that some females can experience nearly all of the symptoms and signs of Fabry disease and may be as severely affected as men [1]. The high heterogeneity of the Fabry phenotype in women has been attributed, at least in part, to random inactivation of one X-chromosome during embryogenesis, which results in a mosaicism of gene expression with some cells expressing the functional enzyme and others expressing the mutated variant [2], [3]. The heterogenic Fabry disease phenotype in women renders both, diagnostic testing and treatment decisions more challenging than in men.

Diagnosis of Fabry disease in adult male patients is based on reduced levels of GLA activity in plasma or leukocytes, and confirmation by genetic mutation analysis [4]. However, in heterozygous females GLA assays can be inconclusive, showing ranges of activity between low to normal levels [5]. Thus, diagnosis of Fabry disease in women requires mutation analysis based on direct sequencing of the GLA gene. Although treatment with enzyme replacement therapy (ERT) is recommended by most experts for all affected men [6], [7], there is less agreement on which female patients should be treated [8]. While treating all heterozygous females is not justified given the benign disease course in many individuals and the high cost of ERT, limiting ERT to patients with end organ damage would delay therapy to a point where irreversible damage has already occurred. Lack of a reliable disease biomarker further hampers individualized therapy and current dosing recommendations for ERT are relatively arbitrary, both in men and in women.

Despite continuous efforts to identify Fabry disease biomarkers, there is still no clearly useful marker. Gb_3_ accumulation in tissues and body fluids (urine and plasma) is one candidate biomarker that has been used for screening [9] and monitoring response to ERT [10], [11]. However, the usefulness of plasma and urinary Gb_3_ levels has been questioned [12], and in particular, it was not elevated in most hemizygous female patients. Thus, reliable biomarkers for Fabry disease are needed, particularly for female patients.

In our study, we used a proteomic approach to identify biomarkers in heterozygous adult female Fabry patients. Capillary electrophoresis coupled to mass spectrometry (CE-MS) is a reliable high-throughput method to simultaneously measure the excretion of hundreds of polypeptides and small proteins in the urine [13]. This technique utilizes capillary electrophoresis (CE) to separate small proteins according to their electrophoretic characteristics, directly followed by mass spectrometric analysis (MS) by electron spray ionization (ESI) and time-of-flight mass spectrometry (TOF-MS). Thus, every detected peptide will be unambiguously characterized by the migration time in CE and the molecular mass determined by MS. CE-MS is characterized by a low amount of sample required, a short analysis time and a high reproducibility owing to the constant buffer composition and flow characteristics of CE that allow stable ESI conditions for coupling to MS throughout the run. Using CE-MS, a total of over 100,000 different peptides have been detected in human urine, and 5,000 of those are detectable in at least 20% of urine samples [14], rendering CE-MS based urine proteomic analysis a powerful tool for the discovery of potential biomarkers. Here, we used CE-MS of urine to identify a proteomic pattern that characterizes female Fabry patients.

## Results

### Patient characteristics

We examined a total of 52 treatment-naïve and 11 ERT treated adult female Fabry patients from three clinical centers. The untreated patients were randomly divided in a 2∶1 ratio into a training cohort and a validation cohort. Clinical characteristics of these two cohorts and the patients under ERT are given in **Table 1**. Patient characteristics did not differ among centers (data not shown). Overall, patients had relatively few symptoms and well preserved renal and cardiac functions. The Mainz Severity Score Index (MSSI) [15] was available for 19 patients of the training cohort (median 9.0, range 0–24) and 8 patients of the validation cohort (median 6.5, range 1–16).

**Table 1:** Clinical characteristics of all studied female Fabry patients.

cohort	training	validation	ERT
N	35	17	11
age (years)	40.9±12.6	35.7±12.8	36.4±16.4
**renal manifestations**			
GFR (ml/min/1.73 m^2^)	87±23	91±13	85±20
urine protein (mg/g crea), median (range)	65 (20–1364)	59 (44–1248)	55 (50–195)
urine albumin (mg/g crea), median (range)	14 (3–864)	10 (5–258)	8 (5–106)
microalbuminuria, N (%)	7 (21%)	4 (24%)	1 (9%)
macroalbuminuria, N (%)	3 (9%)	0 (0%)	0 (0%)
**cardiac manifestations**			
LVMI (g/m^2^)	83±29	81±19	111±77
LVH, N (%)	5 (14%)	1 (6%)	3 (27%)
arrhythmia, N (%)	1 (3%)	0 (0%)	0 (0%)
**nervous system involvement**			
stroke, N (%)	2 (6%)	0 (0%)	2 (18%)
acroparesthesia, N (%)	13 (41%)	8 (47%)	11 (100%)
**other manifestations**			
angiokeratoma, N (%)	4 (13%)	2 (12%)	1 (9%)
hypohydrosis, N (%)	6 (19%)	1 (6%)	4 (36%)

ERT, enzyme replacement therapy; GFR, glomerular filtration rate; LVMI, left ventricular mass index; LVH, left ventricular hypertrophy (defined as LVMI≥110 g/m^2^). Data are mean ± SD, unless otherwise stated.

### Fabry disease is characterized by a unique urinary biomarker profile

**Figure 1:**
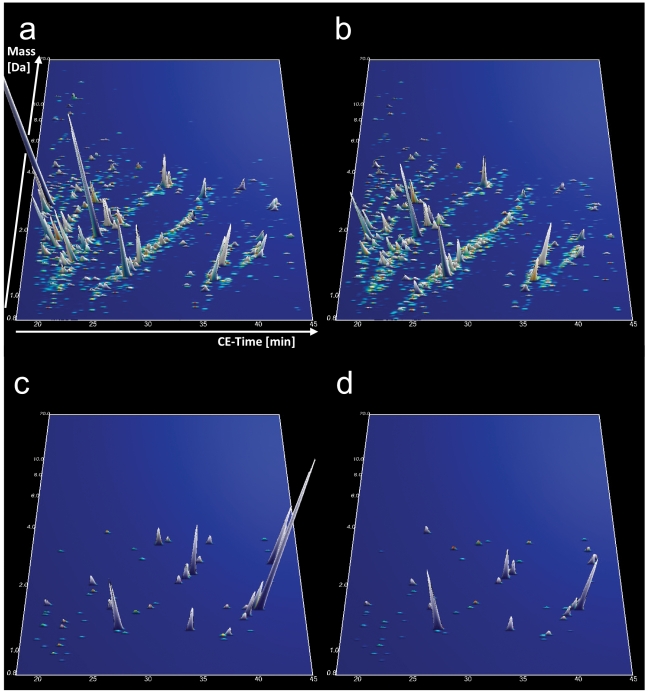
Compiled urinary protein profiles of female Fabry patients (a) and healthy controls (b) included in the training cohort. Normalized MS molecular weight (800–20,000 Da) in logarithmic scale is plotted against normalized CE migration time (18–45 min). The mean signal intensity of polypeptides is given as peak height. 3-D contour plots of the 64 diagnostic markers in the Fabry (c) and healthy control (d) patient cohort with 5× zoom compared to (a) and (b).

**Figure 2:**
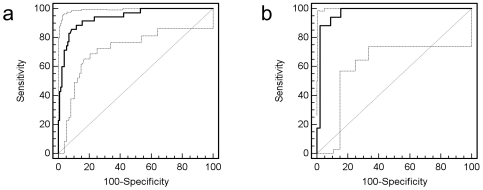
ROC curves for differentiation of Fabry female patients and female healthy controls in the training set upon complete take-one-out crossvalidation (a) and in the independent validation set (b).

The compiled data of the CE-MS analysis of all urine samples from the 35 untreated female Fabry patients and the 89 age-matched female healthy controls of the training cohort are shown in **Figure 1 a and b**, respectively. The comparison of the abundance of individual urinary peptides between patients and controls resulted in the identification of 152 peptides with significantly altered urinary excretion (adjusted p-value<0.05). The CE-MS characteristics of these markers and their regulation in Fabry disease are given in **Table S1**. Due to the fact that the number of potential biomarkers exceeded the number of samples studied, we attempted to identify the most consistent markers by randomly excluding 30% of all patients and controls from the analysis. After repeating this analysis 30 times as an iterative algorithm, we chose only those 64 markers that significantly differed between patients and controls in at least 50% of all permutations. Based on these 64 markers (**Figure 1 c and d**) we next created a support-vector-machine (SVM)-based model to distinguish Fabry patients from controls. The SVM-based model combines the amplitude of all 64 markers for a given urine sample into a score, which denotes the distance of that sample in a 64-dimensional space (every dimension representing the amplitude of one marker) from a hyperplane that is designed to separate the cases from controls. This model correctly classifies all cases and controls in the training cohort, corresponding to an area under the receiver-operator-characteristics curve (AUC) of 1. Complete take-one-out crossvalidation in the training cohort yielded an AUC of 0.939 (95% CI 0.882–0.974) (**Figure 2 a**). We then tested the diagnostic model in an independent validation cohort consisting of 17 untreated female Fabry patients and 45 age- and sex-matched healthy controls. Using as a diagnostic cut-off value the mean between the highest and lowest possible cut-off values that would yield 100% sensitivity and 100% specificity in the training cohort, the model achieved a sensitivity of 88.2% and a specificity of 97.8% in the validation cohort (AUC 0.970, 95% CI 0.891–0.996) (**Figure 2 b**) using genetic mutation analysis as a reference. The two Fabry patients from the validation cohort with false-negative proteomic test results did not obviously differ from the remaining patients, in particular they had both GLA mutations which were not unique in the cohort (one missense and one nonsense mutation).

To further evaluate the specificity of the biomarker model for Fabry disease as compared to other disorders, we applied it to a total of 412 previously analyzed urine probes from female patients suffering from a wide variety of renal, metabolic and cardiovascular diseases. The overall specificity of the model applied to these 412 patients was 97%, i.e. the rate of false positive results for Fabry disease among patients with other diseases was very low (**Table 2**).

**Table 2:** Specificity of the biomarker model for differentiating Fabry disease from other renal, metabolic and cardiovascular diseases.

Disease	Age	False positive/total N	Specificity (%)
ADPKD	31±8	6/78	92
diabetic nephropathy	58±13	0/47	100
FSGS	42±23	0/27	100
heart failure	68±10	1/9	89
hypertension	66±11	1/17	94
IgA nephropathy	35±13	0/24	100
cardiovascular disease	66±7	3/47	94
minimal change disease	42±10	0/12	100
membranous nephropathy	54±20	0/9	100
kidney stones	56±8	0/8	100
renal cell carcinoma	64±11	0/42	100
bladder cancer	66±11	4/46	91
systemic lupus erythematodes	41±11	0/19	100
ANCA vasculitis	67±6	0/27	100

ADPKD, autosomal dominant polycystic kidney disease; FSGS, focal segmental glomerulosclerosis; ANCA, anti-neutrophil cytoplasmic antibodies. Data are mean ± SD, unless otherwise stated.

To gain insight into pathophysiologic mechanisms, we attempted to identify the peptides with altered excretion in Fabry disease. Because the small sample volume used for capillary electrophoresis is not usually sufficient for tandem mass spectrometry based sequencing, we used liquid chromatography-tandem mass spectrometry (LC-MS/MS) for peptide sequencing. We were able to identify the amino acid sequence of 50 out of all 152 differentially excreted peptides, and among the 64 markers used in the diagnostic model, 13 could be identified. The peptide sequences of all identified markers are given along with their CE-MS characteristics in **Table S1**. The majority of identified regulated peptides were collagen fragments with 2/3 of them being up- and 1/3 being downregulated. Interestingly, the C-terminal sequences PPG and PGP were very frequent among the upregulated fragments (6/24 and 11/24, respectively), whereas they were hardly present among the downregulated (0/12 and 1/12, respectively).

### Correlation of biomarkers with disease severity and progression

We wondered whether the proteomic changes could reflect disease severity and predict progression in addition to a potential diagnostic use. We therefore correlated the intensity of all 64 diagnostic biomarkers with GFR, albuminuria, and left ventricular mass index (LVMI) in the total treatment-naïve study population consisting of the training and validation cohorts. As a measure of disease progression, we calculated GFR slope by regressing estimated GFR over time for every patient. Follow-up serum creatinine measurements were available for 36 patients. In these 36 patients, an average of 3.6 (range: 2–7) creatinine measurements were available over a mean period of 3.5±1.9 years (1.8±1.7 years before and 1.7±1.4 years after urine sampling for CE-MS analysis). In the unadjusted analysis, several markers showed significant correlations with these clinical measures of disease severity (6 with GFR, 6 with albuminuria, and 11 with LVMI). However, after adjustment for multiple testing, only two correlations remained significant (both with GFR).

### Effects of enzyme replacement therapy on the biomarker profile

To analyze the effects of enzyme replacement therapy on the urine proteome in Fabry disease, we analyzed spot urine samples of 11 female Fabry patients that were receiving ERT. The clinical characteristics of these patients are summarized in **Table 1**. Notably, the treated patients did not differ significantly in terms of their clinical parameters from the untreated patients except for a higher rate of reported acroparesthesia (p<0.001). In particular, GFR, albuminuria and LVMI were not significantly different. Their median MSSI was 11 (range 5–30) and tended to be higher than in the untreated patients. Details on the type of ERT product, duration and dose, and timing of urine sampling relative to ERT are given in **Table 3**.

**Table 3:** Duration, dose and timing of ERT in the analyzed treated patients.

age (years)	body weight (kg)	ERT product and dose/2 weeks	interval from last ERT dose to urine sampling (days)	treatment duration (years)	biomarker score
62	72	agalsidase alfa 0.22 mg/kg	13	0.6	−1.999 (neg)
28	60	agalsidase alfa 0.18 mg/kg	1	3.6	−1.436 (neg)
25	49	agalsidase alfa 0.21 mg/kg	7	3.8	−1.129 (neg)
17	51	agalsidase alfa 0.20 mg/kg	13	2.4	−0.601 (neg)
43	51	agalsidase alfa 0.28 mg/kg	5	1.1	−0.563 (neg)
59	52	agalsidase alfa 0.20 mg/kg	2	5.6	−0.313 (neg)
22	59	agalsidase alfa 0.20 mg/kg	7	5.6	−0.245 (neg)
34	63	agalsidase alfa 0.22 mg/kg	6	0.1	0.060 (neg)
33	55	agalsidase beta 1.27 mg/kg	13	6.4	0.241 (pos)
20	45	agalsidase alfa 0.20 mg/kg	1	5.9	0.316 (pos)
57	58	agalsidase alfa 0.12 mg/kg	17	2.3	0.455 (pos)

ERT, enzyme replacement therapy. The diagnostic cut off value for the biomarker score is 0.1.

**Figure 3:**
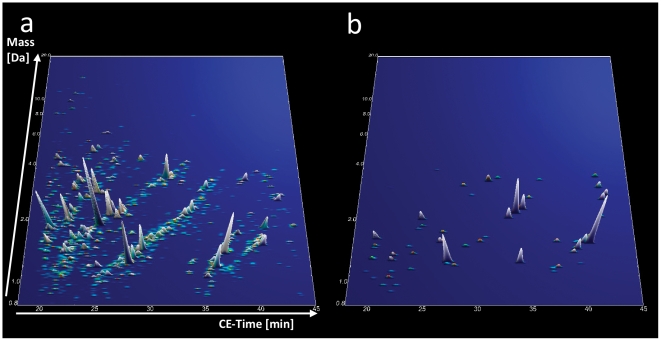
Compiled urinary protein profiles of ERT treated female Fabry patients (a). Normalized MS molecular weight (800–20,000 Da) in logarithmic scale is plotted against normalized CE migration time (18–45 min). The mean signal intensity of polypeptides is given as peak height. (b) 3-D contour plots of the 64 specific markers in the treated Fabry cohort with 5× zoom compared to (a). Note that the proteomic pattern resembles more that of healthy controls (Figure 1b and d) than that of untreated female Fabry patients (Figure 1a and c).

The compiled proteomic data of all treated patients are graphically depicted in **Figure 3 a**. Of the 64 biomarkers (**Figure 3 b**) from the diagnostic model, 31 differed significantly between treated and untreated (training and validation cohort) patients in the unadjusted analysis and 16 remained significant after adjustment for multiple testing. The mean levels of all of these biomarkers were changed toward their mean levels in healthy control subjects. Importantly, when applying the diagnostic biomarker model to the treated patients, only three out of 11 scored positive. Thus, ERT seams to have a profound effect on the urinary proteome, changing it toward that of healthy controls. The 3 ERT treated patients who still scored positive for Fabry disease did not significantly differ from the remaining 8 in terms of treatment duration (4.1±2.1 vs. 3.4±2.3 years, p = 0.557) or time interval between the last ERT infusion and urine sampling for proteomic analysis (7.0±4.4 vs. 10.0±8.3 days, p = 0.364), although one of them received only a reduced dose of ERT.

## Discussion

We have identified and validated a distinct peptide profile in the urine that characterizes adult female Fabry patients. This pattern distinguishes female Fabry patients from healthy controls and from patients with various other forms of kidney or systemic diseases and responds to ERT.

Until now, no study demonstrated the usefulness of a biomarker in adult female Fabry patients as diagnostic marker or as a surrogate marker for disease severity and ERT response. Proteomic techniques offer an unbiased approach to discover unanticipated biomarkers. In addition, the simultaneous detection of hundreds of polypeptides allows for the definition of proteomic biomarker patterns (proteomic profiling), rather than single biomarkers. We used urine rather than serum or plasma for proteomic analysis due to several advantages of urine as sample source that have been discussed in detail elsewhere [16], [17]: First, blood contains a high dynamic range between low-level and highly abundant proteins. Removal of highly abundant proteins, such as albumin, which would otherwise obscure the detection of low-level proteins, leads to a concommitant loss of other proteins due to their binding to albumin or unspecific interaction with the affinity column. Second, urine is relatively stable in its composition if handled properly, which may be in part explained by the completion of endogenous proteolysis at the time of urine voidance. Blood, in contrast, contains high level of endogenous protease activity. Third, urine normally does not contain relevant amounts of cellular elements, obviating the need for preanalytical separation. Finally, urine is enriched in low-molecular-weight proteins and peptides, which can be transferred without an initial protease digestion step directly to protein mass spectrometry (top-down MS). Compared to other methods of proteomic analysis, such as 2-dimensional gel electrophoresis (2DGE) or liquid chromatography (LC) followed by mass spectrometry, CE-MS has the advantage of robustness, high reproducibility, low sample volume required, and short analysis time, which make it particularly suitable for clinical proteomic profiling.

Two studies have been previously published that used a proteomic approach to biomarker discovery in Fabry disease. Moore et al. [18] compared serum samples of 12 male and 1 female pediatric Fabry patients before and after 6 months of ERT by LC-MS/MS and found 5 of 50 identified proteins to be significantly altered under therapy. Vojtova et al. [19] compared 11 male and 9 female Fabry patients with or without ERT to 10 healthy controls using 2DGE followed by MALDI-TOF/TOF and identified 5 differentially expressed proteins. However, none of the above mentioned studies validated their results in an independent cohort. Given the large number of peptides being simultaneously detected using proteomic techniques, vigorous adjustment for multiple testing and validation of the results in an independent cohort are of particular importance [20]. Our study is, to our knowledge, the first proteomic study in Fabry disease, which compares both, treatment-naïve and ERT treated patients to healthy controls and validated the results in an independent cohort.

The biomarker model that we describe distinguishes adult untreated female Fabry patients not only from age-matched healthy controls but also from a large number of different renal diseases with a high degree of specificity. This makes CE-MS particularly useful as a noninvasive diagnostic screening test in unexplained renal, cardiac or cerebrovascular disease. Several recent studies have shown a high prevalence of Fabry disease in populations with unexplained renal failure [21], [22], [23], [24], stroke [25], [26], [27] or hypertrophic cardiomyopathy [28], [29], [30]. However, screening these populations for Fabry disease is hampered by the low sensitivity of GLA activity measurement in female patients (50% [31]–67% [32]) whereas diagnostic sequencing of the GLA gene is not feasible given the high cost (currently ca. 2,000–3,000 USD). CE-MS is available for clinical use and considerably cheaper than genetic testing (currently ca. 500 USD, i.e. similar to the price of GLA activity assays). In terms of diagnostic accuracy, CE-MS also favourably compares to urinary Gb_3_, for which an AUC of 0.876 has been reported [33] (vs. 0.970 for CE-MS in our study), although lyso-Gb_3_ seems to perform better than total Gb_3_ [34]. In addition, because similarly accurate CE-MS based diagnostic models have been developed for a variety of other renal and cardiovascular diseases [13], [35], [36], [37], [38], [39], CE-MS may give hints to an alternative diagnosis in Fabry negative patients, i.e. this approach may offer an efficient diagnostic method, which can detect a variety of diseases using a single diagnostic test. With 97.8%, specificity was high, thus reducing the false positive rate if screening preselected patients. Certainly, the specificity is not ideal, and as a consequence, to avoid high false positive rates, CE-MS is not suitable for screening women at very low risk for Fabry disease, e.g. the general population. Also, mutation analysis will be required for diagnostic confirmation, in particular before initiating a costly therapy. In summary, CE-MS as a diagnostic tool for Fabry disease in females may be clinically useful primarily in the evaluation of patients with unexplained renal disease, hypertrophic cardiomyopathy or cerebrovascular disease, followed by mutation analysis for patients scoring positive in CE-MS.

Importantly, most ERT treated female Fabry patients scored negative in the diagnostic model. Thus, ERT seems to reduce the Fabry-specific alterations in the urinary polypeptide pattern to a level below the diagnostic threshold in most patients receiving ERT. Hence, CE-MS could be useful for monitoring response to treatment, i.e. lack of the urine proteome to normalize may be an indicator of insufficient dosing of ERT. This finding is noteworthy, as ERT dosing recommendations are largely arbitrary to date. Further studies are needed to determine whether the response of the urine proteomic profile to ERT is of prognostic value and whether it is dose-dependent. Given that treated and untreated patients were similar with respect to their end-organ manifestations, reversibility of proteomic alterations under therapy indicates that these alterations probably reflect ongoing pathophysiological processes rather than established organ damage. Thus, it is likely that the changes in the urine proteome that we identified are both dose-dependent and may be of prognostic value as they mirror disease activity.

We found correlations with disease severity for only a minority of the diagnostic biomarkers, which may be explained by a number of reasons: *First*, the diagnostic markers were selected with the aim to achieve a high sensitivity and specificity. Thus, only robust markers that were altered in most patients have been included in the model and these markers may not be the most useful for a severity score. *Second*, most included patients had few manifestations of Fabry disease and the study cohort did not cover the whole spectrum of disease severity. *Third*, most variables used to assess disease severity may be affected by factors other than Fabry disease and show a considerable variation even in healthy subjects. *Fourth*, clinical measures of disease severity mostly reflect irreversible organ damage, whereas many diagnostic pattern biomarkers, as stated above, responded to treatment and thus did not reflect irreversible organ damage. Further studies using larger patient cohorts, including a broader spectrum of disease severity as well as longer follow up time, will be needed to define prognostic markers and to generate a prognostic biomarker model.

Sequencing of naturally occurring peptides by MS/MS still represents a challenge [17]. We were able to identify nearly one third of differentially excreted peptides. Similar to other diseases that have been studied using CE-MS [35], [36], [38], [39], [40], most diagnostic biomarkers identified represent collagen fragments. Indeed, collagen fragments appear to be the major constituents of urinary peptides identified to date [39]. The predominance of collagen fragments among identified urinary peptides may be somewhat biased due to the fact that they are more easily fragmented and detected by MS/MS owing to their high content in proline residues. Nevertheless, urinary collagen fragments likely reflect a high normal physiological turnover of the extracellular matrix that may be altered in disease. Of note, most collagen fragments upregulated in female Fabry disease exhibited one of two characteristic C-terminal motivs, PPG or PGP. This particular pattern seems relatively specific for Fabry disease. Further study is needed to test whether these fragments arise from cleavage by a particular type of protease. It is tempting to speculate that lysosomal proteases, such as cathepsins, are released in Fabry disease due to lysosomal accumulation of Gb_3_ and lead to cleavage of collagen. Among the other identified peptides, 6 were uromodulin fragments, all of which were upregulated. Interestingly, these were all C-terminal fragments. It has previously been shown that Fabry patients excrete reduced amounts of full-length uromodulin but abnormally processed uromodulin lacking the C-terminus [41]. Efforts to sequence the remaining urinary polypeptides are ongoing and may in the future provide additional pathopysiological insights.

We have limited the analysis to adult female Fabry patients, because we felt that biomarkers to guide diagnosis and treatment decisions are particularly needed for in this patient subgroup. Furthermore, we focused on Fabry patients with relatively few manifestations, aiming to identify markers of early disease progression. Future work will address the use of urine proteomics in both, adult male Fabry patients and in children.

In summary, we defined a urinary biomarker model that allows diagnostic evaluation of female patients for Fabry disease with high accuracy and might be used to monitor response to ERT. Further study is needed to identify prognostic markers and to establish a dose-response relationship between ERT and urinary peptide changes.

## Methods

### Ethics Statement

Collection of patient data and collection, storage and analysis of urine samples have been approved by the local ethics committees of the three participating centers (Kantonale Ethikkommission Zürich, Ethik-Kommission bei der Medizinischen Fakultät der Universität Würzburg, and Royal free Hospital and Medical School research ethics committee, respectively). All participating subjects gave written informed consent. This study was performed in accordance with the Helsinki Declaration.

### Patients and Procedures

52 treatment-naïve and 11 ERT-treated female Fabry patients from three clinical centers were studied. The diagnosis of Fabry disease was confirmed by mutation analysis in all patients. Control urine samples were taken from healthy volunteers and patients with a variety of other renal, metabolic and cardiovascular diseases. These samples have been previously collected as part of several clinical studies (refs [13], [35], [36], [37], [38], [39] and as yet unpublished studies). All samples were collected in the morning from midstream urine, frozen at −20°C without prior centrifugation and without addition of protease inhibitors, as suggested in recently published recommendations [42].

### Sample preparation and CE-MS analysis

All urine samples for CE-MS analyses were stored in accordance with current recommendations of EuroKUP and HUPO/HKUPP (http://www.eurokup.org/sites/default/files/StandardProtocolforUrineCollection.pdf) at −20°C until analysis and underwent a maximum of 2 freeze/thaw cycles. A 0.7 mL aliquot was thawed immediately before use, diluted with 0.7 mL of 2 M urea, 10 mM NH_4_OH and 0.02% SDS, filtered using Centrisart ultracentrifugation filter devices (20 kDa MWCO; Sartorius, Goettingen, Germany) at 3,000 g until 1.1 ml of filtrate was obtained and desalted on a PD-10 desalting column (Amersham Bioscience, Uppsala, Sweden) equilibrated in 0.01% NH_4_OH in HPLC-grade H_2_O. Finally, all samples were lyophilized, stored at 4°C, and resuspended in HPLC-grade H_2_O shortly before CE-MS analysis.

CE-MS analysis was performed as described previously [13], [43] using a P/ACE MDQ capillary electrophoresis system (Beckman Coulter, Fullerton, USA) on-line coupled to a Micro-TOF MS (Bruker Daltonic, Bremen, Germany). The ESI sprayer (Agilent Technologies, Palo Alto, CA, USA) was grounded, and the ion spray interface potential was set between −4 and −4.5 kV. Data acquisition and MS acquisition methods were automatically controlled by the CE *via* contact-close-relays. Spectra were accumulated every 3 s, over a range of *m/z* 350 to 3000. Accuracy, precision, selectivity, sensitivity, reproducibility, and stability of the analytical platform were demonstrated and described in great detail elsewhere [13].

### Data processing, cluster analysis and statistical methods

Mass spectral ion peaks representing identical molecules at different charge states were deconvoluted into single masses using MosaiquesVisu software [44]. Migration time and ion signal intensity (amplitude) were normalized using internal polypeptide standards [45], [46]. For the identification of potential biomarkers, the reported p-values were calculated using the Wilcoxon Rank-Sum test followed by adjustment for multiple testing using the method described by Benjamini and Hochberg [47]. Disease-specific polypeptide patterns were generated using SVM based MosaCluster software [48]. Sensitivity and specificity were calculated based on tabulating the number of correctly classified samples. Confidence intervals (95% CI) based on exact binomial calculations were carried out in MedCalc version 8.1.1.0 (MedCalc Software, Mariakerke, Belgium, http://www.medcalc.be). The Receiver Operating Characteristic (ROC) plot was obtained by plotting all sensitivity values on the y-axis against their equivalent 1-specificity values on the x-axis for all available thresholds (MedCalc Software). The AUC was evaluated, as it provides a single measure of overall accuracy that is not dependent upon a particular threshold [49]. To correlate individual biomarkers with measures of disease severity or with response to treatment, Kendall Tau and Mann-Whiney U-test were used, respectively, and adjustment for multiple testing was done using the Bonferroni correction.

### Sequencing of polypeptides

Some of the candidate biomarkers have been previously sequenced using liquid chromatography coupled to tandem mass spectrometry (LC-MS/MS) analysis as recently described in detail [50]. We further tried to identify as yet unidentified urinary peptides that were regulated in Fabry disease. ESI LTQ-OT MS using HCD (higher collisional dissociation) was performed on a LTQ Orbitrap XL equipped with a NanoAcquity system from Waters. Peptides were trapped on a home-made 5 µm 200 Å Magic C18 AQ (Michrom) 0.1×20 mm pre-column and separated on a home-made 5 µm 100 Å Magic C18 AQ (Michrom) 0.75×150 mm column with a gravity-pulled emitter. The analytical separation was run for 65 min using a gradient of H2O/FA 99.9%/0.1% (solvent A) and CH3CN/FA 99.9%/0.1% (solvent B) as follows: 0–1 min 95% A and 5% B, then to 65% A and 35% B at 55 min, and 20% A and 80% B at 65 min at a flow rate of 220 nL/min. For MS survey scans, the OT resolution was set to 60000 and the ion population was set to 5E5 with an m/z window from 400 to 2000. Three precursor ions were selected for collision-induced dissociation in the supplementary hexapole prior to Orbitrap analysis. For this, the ion population was set to 2E5, with an isolation width of 2.5 *m/z* units. The normalized collision energies were set to 40%. Spectral data was converted into .dta files (RAW files generated by ion traps from Thermo Fisher Scientific) and searched against human entries in the Swiss-Prot database (Swiss-Prot Number 2010.06) using the Open Mass Spectrometry Search Algorithm (OMSSA; free from NCBI, http://pubchem.ncbi.nlm.nih.gov/omssa/); e-value cut-off was 0.01. All matched sequences were manually validated.

## Supporting Information

Table S1 **CE-MS characteristics of all urinary peptides that significantly differed between female Fabry patients and controls with sequence information of identified peptides; peptides that were used in the diagnostic biomarker model are depicted in bold.**
(DOCX)

## References

[1] Wilcox WR, Oliveira JP, Hopkin RJ, Ortiz A, Banikazemi M (2008). Females with Fabry disease frequently have major organ involvement: lessons from the Fabry Registry.. Mol Genet Metab.

[2] Dobyns WB, Filauro A, Tomson BN, Chan AS, Ho AW (2004). Inheritance of most X-linked traits is not dominant or recessive, just X-linked.. Am J Med Genet A.

[3] Guffon N (2003). Clinical presentation in female patients with Fabry disease.. J Med Genet.

[4] Desnick RJ, Brady R, Barranger J, Collins AJ, Germain DP (2003). Fabry disease, an under-recognized multisystemic disorder: expert recommendations for diagnosis, management, and enzyme replacement therapy.. Ann Intern Med.

[5] Andrade J, Waters PJ, Singh RS, Levin A, Toh BC (2008). Screening for Fabry disease in patients with chronic kidney disease: limitations of plasma alpha-galactosidase assay as a screening test.. Clin J Am Soc Nephrol.

[6] Eng CM, Guffon N, Wilcox WR, Germain DP, Lee P (2001). Safety and efficacy of recombinant human alpha-galactosidase A–replacement therapy in Fabry's disease.. N Engl J Med.

[7] Schiffmann R, Kopp JB, Austin HA, Sabnis S, Moore DF (2001). Enzyme replacement therapy in Fabry disease: a randomized controlled trial.. Jama.

[8] El Dib RP, Pastores GM (2010). Enzyme replacement therapy for Anderson-Fabry disease.. Cochrane Database Syst Rev.

[9] Kitagawa T, Ishige N, Suzuki K, Owada M, Ohashi T (2005). Non-invasive screening method for Fabry disease by measuring globotriaosylceramide in whole urine samples using tandem mass spectrometry.. Mol Genet Metab.

[10] Rozenfeld PA, De Francesco NP, Borrajo GJ, Ceci R, Fossati CA (2009). An easy and sensitive method for determination of globotriaosylceramide (Gb3) from urinary sediment: utility for Fabry disease diagnosis and treatment monitoring.. Clin Chim Acta.

[11] Whitfield PD, Calvin J, Hogg S, O'Driscoll E, Halsall D (2005). Monitoring enzyme replacement therapy in Fabry disease–role of urine globotriaosylceramide.. J Inherit Metab Dis.

[12] Young E, Mills K, Morris P, Vellodi A, Lee P (2005). Is globotriaosylceramide a useful biomarker in Fabry disease?. Acta Paediatr Suppl.

[13] Theodorescu D, Wittke S, Ross MM, Walden M, Conaway M (2006). Discovery and validation of new protein biomarkers for urothelial cancer: a prospective analysis.. Lancet Oncol.

[14] Coon JJ, Zurbig P, Dakna M, Dominiczak AF, Decramer S (2008). CE-MS analysis of the human urinary proteome for biomarker discovery and disease diagnostics.. Proteomics Clin Appl.

[15] Whybra C, Kampmann C, Krummenauer F, Ries M, Mengel E (2004). The Mainz Severity Score Index: a new instrument for quantifying the Anderson-Fabry disease phenotype, and the response of patients to enzyme replacement therapy.. Clin Genet.

[16] Metzger J, Luppa PB, Good DM, Mischak H (2009). Adapting mass spectrometry-based platforms for clinical proteomics applications: The capillary electrophoresis coupled mass spectrometry paradigm.. Crit Rev Clin Lab Sci.

[17] Mischak H, Coon JJ, Novak J, Weissinger EM, Schanstra JP (2009). Capillary electrophoresis-mass spectrometry as a powerful tool in biomarker discovery and clinical diagnosis: an update of recent developments.. Mass Spectrom Rev.

[18] Moore DF, Krokhin OV, Beavis RC, Ries M, Robinson C (2007). Proteomics of specific treatment-related alterations in Fabry disease: a strategy to identify biological abnormalities.. Proc Natl Acad Sci U S A.

[19] Vojtova L, Zima T, Tesar V, Michalova J, Prikryl P (2010). Study of urinary proteomes in Anderson-Fabry disease.. Ren Fail.

[20] Mischak H, Apweiler R, Banks R, Conaway M, Coon J (2007). Clinical Proteomics: a need to define the field and to begin to set adequate standards.. Proteomics Clin Appl.

[21] Kotanko P, Kramar R, Devrnja D, Paschke E, Voigtlander T (2004). Results of a nationwide screening for Anderson-Fabry disease among dialysis patients.. J Am Soc Nephrol.

[22] Tanaka M, Ohashi T, Kobayashi M, Eto Y, Miyamura N (2005). Identification of Fabry's disease by the screening of alpha-galactosidase A activity in male and female hemodialysis patients.. Clin Nephrol.

[23] Merta M, Reiterova J, Ledvinova J, Poupetova H, Dobrovolny R (2007). A nationwide blood spot screening study for Fabry disease in the Czech Republic haemodialysis patient population.. Nephrol Dial Transplant.

[24] Gaspar P, Herrera J, Rodrigues D, Cerezo S, Delgado R (2010). Frequency of Fabry disease in male and female haemodialysis patients in Spain.. BMC Med Genet.

[25] Rolfs A, Bottcher T, Zschiesche M, Morris P, Winchester B (2005). Prevalence of Fabry disease in patients with cryptogenic stroke: a prospective study.. Lancet.

[26] Baptista MV, Ferreira S, Pinho EMT, Carvalho M, Cruz VT (2010). Mutations of the GLA gene in young patients with stroke: the PORTYSTROKE study–screening genetic conditions in Portuguese young stroke patients.. Stroke.

[27] Brouns R, Thijs V, Eyskens F, Van den Broeck M, Belachew S (2010). Belgian Fabry study: prevalence of Fabry disease in a cohort of 1000 young patients with cerebrovascular disease.. Stroke.

[28] Nakao S, Takenaka T, Maeda M, Kodama C, Tanaka A (1995). An atypical variant of Fabry's disease in men with left ventricular hypertrophy.. N Engl J Med.

[29] Sachdev B, Takenaka T, Teraguchi H, Tei C, Lee P (2002). Prevalence of Anderson-Fabry disease in male patients with late onset hypertrophic cardiomyopathy.. Circulation.

[30] Chimenti C, Pieroni M, Morgante E, Antuzzi D, Russo A (2004). Prevalence of Fabry disease in female patients with late-onset hypertrophic cardiomyopathy.. Circulation.

[31] Mehta A, Ricci R, Widmer U, Dehout F, Garcia de Lorenzo A (2004). Fabry disease defined: baseline clinical manifestations of 366 patients in the Fabry Outcome Survey.. Eur J Clin Invest.

[32] Linthorst GE, Vedder AC, Aerts JM, Hollak CE (2005). Screening for Fabry disease using whole blood spots fails to identify one-third of female carriers.. Clin Chim Acta.

[33] Paschke E, Fauler G, Winkler H, Schlagenhauf A, Plecko B (2010). Urinary Total Globotriaosylceramide and Isoforms to Identify Women With Fabry Disease: A Diagnostic Test Study.. Am J Kidney Dis.

[34] Auray-Blais C, Ntwari A, Clarke JT, Warnock DG, Oliveira JP (2010). How well does urinary lyso-Gb3 function as a biomarker in Fabry disease?. Clin Chim Acta.

[35] Delles C, Schiffer E, von Zur Muhlen C, Peter K, Rossing P (2010). Urinary proteomic diagnosis of coronary artery disease: identification and clinical validation in 623 individuals.. J Hypertens.

[36] Kistler AD, Mischak H, Poster D, Dakna M, Wuthrich RP (2009). Identification of a unique urinary biomarker profile in patients with autosomal dominant polycystic kidney disease.. Kidney Int.

[37] Haubitz M, Good DM, Woywodt A, Haller H, Rupprecht H (2009). Identification and validation of urinary biomarkers for differential diagnosis and evaluation of therapeutic intervention in anti-neutrophil cytoplasmic antibody-associated vasculitis.. Mol Cell Proteomics.

[38] Schiffer E, Vlahou A, Petrolekas A, Stravodimos K, Tauber R (2009). Prediction of muscle-invasive bladder cancer using urinary proteomics.. Clin Cancer Res.

[39] Good DM, Zurbig P, Argiles A, Bauer HW, Behrens G (2010). Naturally occurring human urinary peptides for use in diagnosis of chronic kidney disease.. Mol Cell Proteomics.

[40] Rossing K, Mischak H, Dakna M, Zurbig P, Novak J (2008). Urinary proteomics in diabetes and CKD.. J Am Soc Nephrol.

[41] Vylet'al P, Hulkova H, Zivna M, Berna L, Novak P (2008). Abnormal expression and processing of uromodulin in Fabry disease reflects tubular cell storage alteration and is reversible by enzyme replacement therapy.. J Inherit Metab Dis.

[42] Thongboonkerd V (2007). Practical points in urinary proteomics.. J Proteome Res.

[43] Wittke S, Mischak H, Walden M, Kolch W, Radler T (2005). Discovery of biomarkers in human urine and cerebrospinal fluid by capillary electrophoresis coupled to mass spectrometry: towards new diagnostic and therapeutic approaches.. Electrophoresis.

[44] Neuhoff N, Kaiser T, Wittke S, Krebs R, Pitt A (2004). Mass spectrometry for the detection of differentially expressed proteins: a comparison of surface-enhanced laser desorption/ionization and capillary electrophoresis/mass spectrometry.. Rapid Commun Mass Spectrom.

[45] Theodorescu D, Fliser D, Wittke S, Mischak H, Krebs R (2005). Pilot study of capillary electrophoresis coupled to mass spectrometry as a tool to define potential prostate cancer biomarkers in urine.. Electrophoresis.

[46] Jantos-Siwy J, Schiffer E, Brand K, Schumann G, Rossing K (2009). Quantitative urinary proteome analysis for biomarker evaluation in chronic kidney disease.. J Proteome Res.

[47] Reiner A, Yekutieli D, Benjamini Y (2003). Identifying differentially expressed genes using false discovery rate controlling procedures.. Bioinformatics.

[48] Decramer S, Wittke S, Mischak H, Zurbig P, Walden M (2006). Predicting the clinical outcome of congenital unilateral ureteropelvic junction obstruction in newborn by urinary proteome analysis.. Nat Med.

[49] DeLeo J

[50] Zurbig P, Renfrow MB, Schiffer E, Novak J, Walden M (2006). Biomarker discovery by CE-MS enables sequence analysis via MS/MS with platform-independent separation.. Electrophoresis.

