# Supplementary material for: A Distinct Urinary Biomarker Pattern Characteristic of Female Fabry Patients That Mirrors Response to Enzyme Replacement Therapy
Source: PLoS One. 2011 Jun 15;6(6):e20534. doi: 10.1371/journal.pone.0020534 (PMC3115947; doi:10.1371/journal.pone.0020534)
Supplement: Table S1 — CE-MS characteristics of all urinary peptides that significantly differed between female Fabry patients and controls with sequence information of identified peptides; peptides that were used in the diagnostic biomarker model are depicted in bold. (DOCX) [file pone.0020534.s001.docx]

**Table S1.** CE-MS characteristics of all urinary peptides that significantly differed between female Fabry patients and controls with sequence information of identified peptides; peptides that were used in the diagnostic biomarker model are depicted in bold.

| **Mass (Da)** | **CE-Time (min)** | **Adjusted P-value** | **Fold change in Fabry** | **Sequence** | **Parental protein name** | **SwissProt/TrEMBLE name** |
| --- | --- | --- | --- | --- | --- | --- |
| **2084.92** | **32.87** | **2.30E-08** | **23.37** |  |  |  |
| **2650.17** | **34.96** | **8.88E-11** | **9.65** |  |  |  |
| **2764.21** | **42.63** | **6.32E-11** | **6.81** |  |  |  |
| **2608.13** | **42.24** | **1.61E-09** | **5.23** |  |  |  |
| **3397.56** | **31.85** | **3.05E-08** | **4.82** |  |  |  |
| **1587.66** | **39.47** | **7.27E-08** | **4.31** |  |  |  |
| **3286.55** | **30.92** | **8.51E-07** | **4.28** |  |  |  |
| **1474.66** | **20.05** | **5.57E-07** | **4.07** |  |  |  |
| 1580.88 | 23.87 | 3.87E-07 | 4.01 | IDQSRVLNLGPITR | Uromodulin | UROM_HUMAN |
| **1138.59** | **19.51** | **2.28E-06** | **3.83** |  |  |  |
| **1247.48** | **39.61** | **3.41E-06** | **3.72** |  |  |  |
| 1705.73 | 40.44 | 3.36E-04 | 3.56 |  |  |  |
| **2340.99** | **33.71** | **3.20E-04** | **3.28** |  |  |  |
| **1632.71** | **40.15** | **4.29E-06** | **3.18** |  |  |  |
| **1505.61** | **28.77** | **4.16E-05** | **3.14** |  |  |  |
| **2320** | **34.29** | **1.03E-07** | **3.13** |  |  |  |
| **3023.41** | **20.95** | **3.36E-04** | **3.08** |  |  |  |
| **2248.97** | **33.69** | **2.28E-06** | **3.04** | **GPAGQDGVGGDKGEDGDPGQpGPpG** | **Collagen alpha-1(XI) chain** | **COBA1_HUMAN** |
| 1679.95 | 23.82 | 9.17E-05 | 2.98 | VIDQSRVLNLGPITR | Uromodulin | UROM_HUMAN |
| **3319.38** | **36.08** | **5.67E-05** | **2.97** |  |  |  |
| **1569.65** | **39.31** | **2.60E-05** | **2.95** |  |  |  |
| 1595.7 | 30.01 | 2.28E-06 | 2.95 | GPpGEAGKpGEQGVpGD | Collagen alpha-1(I) chain | CO1A1_HUMAN |
| **1287.59** | **21.87** | **9.09E-05** | **2.85** | **LGPHAGDVEGHLS** | **Apolipoprotein A-IV** | **APOA4_HUMAN** |
| **1478.61** | **39.3** | **1.43E-03** | **2.85** |  |  |  |
| **1584.51** | **37.5** | **1.33E-02** | **2.81** |  |  |  |
| **1217.48** | **27.77** | **1.18E-04** | **2.74** |  |  |  |
| 1689.74 | 40.6 | 4.29E-06 | 2.7 |  |  |  |
| **1169.57** | **23.71** | **1.89E-04** | **2.68** |  |  |  |
| 2577.25 | 24.67 | 1.61E-05 | 2.67 | DDILASPPRLPEPQPYPGAPHHSS | Collagen alpha-1(XVIII) chain | COIA1_HUMAN |
| **1063.44** | **20.28** | **9.17E-05** | **2.64** |  |  |  |
| **1312.62** | **20.01** | **3.56E-04** | **2.55** |  |  |  |
| 1483.7 | 20.65 | 3.41E-06 | 2.53 |  |  |  |
| 1754.9 | 31.26 | 4.84E-02 | 2.46 | SGSVIDQSRVLNLGPIT | Uromodulin | UROM_HUMAN |
| **1380.64** | **23.83** | **1.11E-03** | **2.41** |  |  |  |
| **1673.76** | **40.72** | **4.16E-05** | **2.37** |  |  |  |
| 3256.53 | 33.03 | 2.34E-04 | 2.35 |  |  |  |
| **1577.69** | **40.08** | **4.27E-05** | **2.34** |  |  |  |
| **1462.62** | **39.42** | **4.01E-05** | **2.32** |  |  |  |
| 3001.43 | 35.4 | 2.36E-05 | 2.31 |  |  |  |
| 1099.5 | 21.67 | 5.14E-04 | 2.29 |  |  |  |
| 3404.62 | 25.94 | 7.01E-04 | 2.29 |  |  |  |
| **2666.18** | **35.03** | **3.26E-03** | **2.28** |  |  |  |
| **1561.69** | **40.63** | **3.87E-07** | **2.25** |  |  |  |
| 2642.21 | 27.7 | 1.28E-03 | 2.23 | QGPpGPSGEEGKRGPNGEAGSAGPpGPpG | Collagen alpha-2(I) chain | CO1A2_HUMAN |
| **1361.63** | **21.99** | **2.31E-04** | **2.22** |  |  |  |
| 1312.62 | 22.46 | 1.13E-04 | 2.2 |  |  |  |
| 1452.66 | 23.61 | 1.08E-03 | 2.18 |  |  |  |
| 1417.64 | 20.03 | 2.36E-05 | 2.14 |  |  |  |
| **1834.84** | **24.14** | **2.98E-03** | **2.1** |  |  |  |
| 1338.6 | 23.99 | 5.64E-04 | 2.03 |  |  |  |
| **1405.61** | **39.04** | **1.22E-04** | **2.02** |  |  |  |
| **1588.71** | **30.15** | **3.68E-03** | **2** | **TGLSMDGGGSPKGDVDP** | **Na/K-ATPase gamma chain** | **ATNG_HUMAN** |
| **1138.47** | **37.07** | **6.93E-04** | **2** | **DGEAGAQGPpGPA** | **Collagen alpha-1(I) chain** | **CO1A1_HUMAN** |
| **1142.56** | **21.89** | **3.26E-03** | **1.97** |  |  |  |
| **1181.48** | **37** | **1.34E-02** | **1.9** |  |  |  |
| **988.52** | **22.44** | **5.67E-05** | **1.88** |  |  |  |
| 1594.73 | 23.05 | 4.76E-03 | 1.87 | ApGGKGDAGApGERGPpG | Collagen alpha-1(III) chain | CO3A1_HUMAN |
| 2007.95 | 22.1 | 4.35E-02 | 1.83 | DGESGRpGRpGERGLpGPpG | Collagen alpha-1(III) chain | CO3A1_HUMAN |
| **923.42** | **22.01** | **2.36E-05** | **1.79** |  |  |  |
| **1195.48** | **37.51** | **1.66E-03** | **1.78** |  |  |  |
| 1447.7 | 19.47 | 7.14E-03 | 1.77 |  |  |  |
| **1892.97** | **24.56** | **6.29E-03** | **1.76** |  |  |  |
| **988.5** | **21.25** | **6.00E-03** | **1.72** | **YQTNKAKH** | **Cystatin-B** | **CYTB_HUMAN** |
| 949.22 | 34.33 | 2.61E-02 | 1.71 |  |  |  |
| 1199.58 | 21.95 | 2.98E-03 | 1.69 |  |  |  |
| 1485.67 | 23.77 | 1.08E-03 | 1.68 | DGQpGAKGEpGDAGAK | Collagen alpha-1(I) chain | CO1A1_HUMAN |
| 3264.56 | 25.75 | 3.80E-02 | 1.68 | AAGEPGkAGERGVpGPpGAVGPAGKDGEAGAQGPPGP | Collagen alpha-1(I) chain | CO1A1_HUMAN |
| 1068.45 | 24.76 | 2.47E-05 | 1.66 |  |  |  |
| 1684.67 | 30.66 | 1.05E-03 | 1.64 |  |  |  |
| 944.51 | 21.25 | 1.18E-03 | 1.64 |  |  |  |
| **2751.34** | **29.23** | **1.34E-03** | **1.64** |  |  |  |
| **1576.6** | **26.37** | **3.85E-04** | **1.63** |  |  |  |
| 2132.91 | 25.83 | 1.25E-02 | 1.62 |  |  |  |
| 1179.52 | 37.49 | 2.28E-02 | 1.62 | GPpGPpGPSSNQG | Collagen alpha-6(IV) chain | CO4A6_HUMAN |
| 1522.7 | 29.41 | 5.58E-03 | 1.61 | SpGSpGPDGKTGPpGPA | Collagen alpha-1(I) chain | CO1A1_HUMAN |
| 1813.72 | 31.69 | 1.34E-02 | 1.61 |  |  |  |
| **1257.44** | **33.92** | **1.18E-03** | **1.6** |  |  |  |
| **1068.51** | **21.75** | **1.43E-03** | **1.59** |  |  |  |
| 1451.66 | 29.17 | 1.48E-05 | 1.57 | SpGSpGPDGKTGPPGp | Collagen alpha-1(I) chain | CO1A1_HUMAN |
| 1040.48 | 25.05 | 4.13E-04 | 1.57 | SpGPDGKTGPp | Collagen alpha-1(I) chain | CO1A1_HUMAN |
| **1510.68** | **20.17** | **1.43E-03** | **1.56** |  |  |  |
| 3292.54 | 39.42 | 3.76E-03 | 1.54 |  |  |  |
| 1255.48 | 35.77 | 2.76E-02 | 1.53 |  |  |  |
| 1782.84 | 25.91 | 4.14E-02 | 1.52 |  |  |  |
| 1299.58 | 22.38 | 2.29E-02 | 1.51 |  |  |  |
| 3143.45 | 32.87 | 2.16E-02 | 1.51 |  |  |  |
| **1638.73** | **20.23** | **1.38E-02** | **1.5** | **AGSEADHEGTHSTKRG** | **Fibrinogen alpha chain** | **FIBA_HUMAN** |
| 1194.55 | 26.7 | 2.29E-02 | 1.49 | SpGPDGKTGPpGP | Collagen alpha-1(I) chain | CO1A1_HUMAN |
| 1153.31 | 35.61 | 2.56E-02 | 1.49 |  |  |  |
| 1911.05 | 24.98 | 2.61E-02 | 1.48 | SGSVIDQSRVLNLGPITR | Uromodulin | UROM_HUMAN |
| **2048.93** | **24.46** | **1.71E-02** | **1.48** |  |  |  |
| **3477.44** | **41.78** | **1.66E-04** | **1.47** |  |  |  |
| 2039.13 | 21.78 | 2.86E-02 | 1.46 | SGSVIDQSRVLNLGPITRK | Uromodulin | UROM_HUMAN |
| 4044.92 | 26.37 | 7.32E-03 | 1.44 |  |  |  |
| 1128.39 | 33.59 | 2.82E-02 | 1.44 | DFDDFNLED | CD99 antigen-like protein 2 | C99L2_HUMAN |
| 2220.99 | 27.13 | 9.00E-03 | 1.43 | ADGQpGAKGEpGDAGAKGDAGPpGP | Collagen alpha-1(I) chain | CO1A1_HUMAN |
| 1767 | 24.11 | 1.28E-03 | 1.41 | SVIDQSRVLNLGPITR | Uromodulin | UROM_HUMAN |
| 1508.68 | 29.33 | 3.90E-02 | 1.4 | GSpGSpGPDGKTGPPGp | Collagen alpha-1(I) chain | CO1A1_HUMAN |
| 1435.66 | 28.84 | 4.42E-02 | 1.38 | SpGSPGPDGKTGPpGP | Collagen alpha-1(I) chain | CO1A1_HUMAN |
| 2175.01 | 33.28 | 3.29E-02 | 1.37 | AGPpGEAGKpGEQGVpGDLGApGP | Collagen alpha-1(I) chain | CO1A1_HUMAN |
| 1032.5 | 21.21 | 1.59E-02 | 1.37 |  |  |  |
| **1240.55** | **27.05** | **4.89E-03** | **1.35** | **FSVMPGLKMTM** | **inter-alpha (globulin) inhibitor H4 isoform 2 precursor** | **ITIH4_HUMAN** |
| 1085.47 | 21.94 | 1.34E-02 | 1.34 |  |  |  |
| **1764.81** | **26.56** | **7.14E-03** | **1.34** |  |  |  |
| 2266.02 | 22.16 | 2.38E-02 | 1.33 | QNGEpGGKGERGAPGEKGEGGppG | Collagen alpha-1(III) chain | CO3A1_HUMAN |
| 2013.91 | 25.19 | 4.00E-02 | 1.3 | NSGEpGApGSKGDTGAKGEpGP | Collagen alpha-1(I) chain | CO1A1_HUMAN |
| 1016.27 | 35.65 | 1.39E-02 | 1.3 |  |  |  |
| 2695.2 | 23.52 | 3.94E-02 | 1.27 | NRGERGSEGSpGHpGQpGppGPPGAPGp | Collagen alpha-1(III) chain | CO3A1_HUMAN |
| 1525.48 | 37.16 | 3.00E-02 | 1.27 |  |  |  |
| 3657.67 | 40.71 | 7.90E-04 | 1.26 |  |  |  |
| **1268.57** | **27.25** | **1.33E-02** | **1.21** | **SpGERGETGPpGP** | **Collagen alpha-1 (III) chain** | **CO3A1_HUMAN** |
| 2377.1 | 20.8 | 3.39E-02 | 1.2 | GKNGDDGEAGKpGRpGERGPpGPQ | Collagen alpha-1(I) chain | CO1A1_HUMAN |
| 2570.19 | 42.56 | 4.31E-02 | 1.16 |  |  |  |
| **2117.03** | **42** | **5.10E-04** | **1.14** |  |  |  |
| 1084.43 | 25.23 | 6.93E-04 | 1.13 |  |  |  |
| 1445.62 | 37.36 | 3.39E-02 | 1.11 | DGVGQpGLPGpPGPpG | Collagen alpha-1(XVIII) chain | COIA1_HUMAN |
| 956.44 | 20.36 | 2.13E-02 | 1.1 |  |  |  |
| 1225.53 | 26.31 | 3.76E-02 | 1.06 |  |  |  |
| 931.47 | 20 | 4.01E-02 | 0.84 |  |  |  |
| **1495.68** | **23.36** | **1.66E-03** | **0.77** |  |  |  |
| 1916.77 | 20.32 | 3.95E-02 | 0.65 |  |  |  |
| 2281.98 | 33.93 | 9.85E-03 | 0.64 | ANGApGNDGAKGDAGApGApGSQGApG | Collagen alpha-1(I) chain | CO1A1_HUMAN |
| 4654.08 | 25.83 | 2.59E-02 | 0.63 |  |  |  |
| 3425.61 | 31.27 | 5.58E-03 | 0.62 | ASHTSDSDVPSGVTEVVVKLFDSDPITVTVPVE | Clusterin | CLUS_HUMAN |
| 1697.74 | 30.88 | 3.47E-02 | 0.61 | NGApGNDGAKGDAGApGApG | Collagen alpha-1(I) chain | CO1A1_HUMAN |
| 3013.29 | 22.29 | 2.16E-02 | 0.6 | ESGREGApGAEGSpGRDGSpGAKGDRGETGPA | Collagen alpha-1(I) chain | CO1A1_HUMAN |
| **1265.59** | **27.09** | **5.58E-03** | **0.58** | **SpGPDGKTGPpGPA** | **Collagen alpha-1 (I) chain** | **CO1A1_HUMAN** |
| 1806.83 | 23.06 | 3.78E-02 | 0.57 |  |  |  |
| 4005.81 | 33.06 | 9.98E-03 | 0.55 |  |  |  |
| **2210.95** | **33.61** | **4.46E-03** | **0.53** | **NGApGNDGAKGDAGApGApGSQGApG** | **Collagen alpha-1 (I) chain** | **CO1A1_HUMAN** |
| **2087.97** | **32.91** | **5.64E-03** | **0.52** | **GPpGEAGkPGEQGVPGDLGApGP** | **Collagen alpha-1 (I) chain** | **CO1A1_HUMAN** |
| **3091.44** | **28.4** | **5.98E-03** | **0.52** |  |  |  |
| 1367.64 | 38.88 | 2.47E-02 | 0.51 | PpGPpGPpGPPGTPV | Collagen alpha-1(XVIII) chain | COIA1_HUMAN |
| 3223.42 | 39.13 | 1.49E-02 | 0.49 |  |  |  |
| 3734.72 | 32.5 | 7.14E-03 | 0.49 |  |  |  |
| **1666.78** | **30.66** | **6.28E-03** | **0.4** | **KpGEQGVpGDLGApGPSG** | **Collagen alpha-1 (I) chain** | **CO1A1_HUMAN** |
| **2339** | **34.01** | **4.91E-03** | **0.4** | **GANGApGNDGAKGDAGApGApGSQGApG** | **Collagen alpha-1(I) chain** | **COL1A1_HUMAN** |
| 1562.69 | 22.46 | 1.12E-02 | 0.38 |  |  |  |
| 3098.44 | 30.06 | 3.11E-02 | 0.38 | LTGNpGVQGPEGKLGPLGApGEDGRpGpPGSIG | Collagen alpha-2(V) chain | CO5A2_HUMAN |
| 911.43 | 25.88 | 5.98E-03 | 0.37 | DGKTGPpGPA | Collagen alpha-1(I) chain | CO1A1_HUMAN |
| **3775.75** | **25.59** | **1.34E-02** | **0.37** |  |  |  |
| 1993.88 | 32.19 | 1.06E-02 | 0.36 |  |  |  |
| 1032.45 | 25.9 | 5.98E-03 | 0.34 |  |  |  |
| 860.36 | 26.14 | 5.08E-03 | 0.34 |  |  |  |
| 911.26 | 34.35 | 2.88E-02 | 0.31 |  |  |  |
| 3290.5 | 24.14 | 1.97E-03 | 0.29 |  |  |  |
| 1297.58 | 27.37 | 5.58E-03 | 0.29 | SpGSpGPDGKTGPp | Collagen alpha-1(I) chain | CO1A1_HUMAN |
| **3035.19** | **42.02** | **1.66E-04** | **0.25** |  |  |  |
| 3589.68 | 25.03 | 1.28E-03 | 0.23 |  |  |  |
| 2080.94 | 20.2 | 1.83E-04 | 0.19 |  |  |  |
| 2816.33 | 28.4 | 5.14E-04 | 0.12 |  |  |  |
| 984.45 | 24.92 | 1.34E-02 | 0.12 | LAADDPEVR | Ephrin-A1 | EFNA1_HUMAN |
